# A Modified Back Propagation Artificial Neural Network Model Based on Genetic Algorithm to Predict the Flow Behavior of 5754 Aluminum Alloy

**DOI:** 10.3390/ma11050855

**Published:** 2018-05-21

**Authors:** Changqing Huang, Xiaodong Jia, Zhiwu Zhang

**Affiliations:** 1 Light Alloy Research Institute, Central South University, Changsha 410083, China; 163811003@csu.edu.cn (X.J.); 163812020@csu.edu.cn (Z.Z.); 2 State Key Laboratory of High-performance Complicated Manufacturing, Central South University, Changsha 410083, China; 3 School of Mechanical and Electrical Engineering, Central South University, Changsha 410083, China

**Keywords:** 5754 aluminum alloy, hot compressive, constitutive model, artificial neural network, genetic algorithm, dynamic softening mechanism

## Abstract

In order to predict flow behavior and find the optimum hot working processing parameters for 5754 aluminum alloy, the experimental flow stress data obtained from the isothermal hot compression tests on a Gleeble-3500 thermo-simulation apparatus, with different strain rates (0.1–10 s^–1^) and temperatures (300–500 °C), were used to construct the constitutive models of the strain-compensation Arrhenius (SA) and back propagation (BP) artificial neural network (ANN). In addition, an optimized BP–ANN model based on the genetic algorithm (GA) was established. Furthermore, the predictability of the three models was evaluated by the statistical indicators, including the correlation coefficient (*R*) and average absolute relative error (*AARE*). The results showed that the R of the SA model, BP–ANN model, and ANN–GA model were 0.9918, 0.9929, and 0.9999, respectively, while the *AARE* of these models was found to be 3.2499–5.6774%, 0.0567–5.4436% and 0.0232–1.0485%, respectively. The prediction error of the SA model was high at 400 °C. It was more accurate to use the BP–ANN model to determine the flow behavior compared to the SA model. However, the BP–ANN model had more instability at 300 °C and a true strain in the range of 0.4–0.6. When compared with the SA model and BP–ANN model, the ANN–GA model had a more efficient and more accurate prediction ability during the whole deformation process. Furthermore, the dynamic softening characteristic was analyzed by the flow curves. All curves showed that 5754 aluminum alloy showed the typical rheological characteristics. The flow stress rose rapidly with increasing strain until it reached a peak. After this, the flow stress remained constant, which demonstrates a steady flow softening phenomenon. Besides, the flow stress and the required variables to reach the steady state deformation increased with increasing strain rate and decreasing temperature.

## 1. Introduction

Hot working is a significant process which directly affects the formability of materials and the mechanical properties of the product [1,2,3,4]. However, in the actual production process of hot compression, the internal structure and properties of the metal will obviously change, resulting in different mechanical behavior in the subsequent deformation. The flow behavior of materials is an important factor that needs to be considered in actual production as it is closely related to the microstructure and properties of the products [5,6]. It reflects the non-linear relationship between the flow stress and control parameters, including strain, strain rate, and temperature of deformation, which is often determined by the constitutive model [7,8,9]. The constitutive model can be employed to uncover the deformation mechanism of materials and to develop the dynamic model of recrystallization. Therefore, the establishment of a high-precision constitutive model for materials has become a focus of researchers.

In the current study, many constitutive models including physical analysis, artificial neural network (ANN), and phenomenological models for different materials have been developed on the basis of hot deformation tests [10,11,12,13]. The physical analysis models have to regress many material coefficients, which inevitably imposes serious constraints on their practical applications [11]. Due to the effective avoidance of contact with the physical mechanism and increased accuracy, the phenomenological constitutive models, such as the strain-compensation Arrhenius (SA) model, require less material constants, and thus, are widely adopted in practice [14,15,16,17]. In order to improve the predictive accuracy, many researchers have modified the phenomenological constitutive models [18,19]. However, when it comes to different experimental data and materials with different processing states, the regression constants of physical analysis and phenomenological models using the regression method have to be recalculated [20,21]. The ANN modeling method, without the specific mathematical model, provides a new idea for the study of the constitutive behavior of materials. This does not require repeated regression calculations, and high-precision predictions can be obtained by the ANN model [22,23,24]. It has a great advantage of being able to deal with complicated and non-linear relationships. Hence, ANN has been widely used to predict the flow behavior of metal materials [25,26,27,28]. Nevertheless, the uncertainty and instability of ANN in predicting flow behavior limits its use. The introduction of genetic algorithm (GA) can remove this limitation, improve its stability and accuracy, and search for the optimal weight and threshold for ANN at the same time.

The 5754 aluminum alloy is a typical Al–Mg alloy, and has moderate strength, favorable corrosion resistance, good weldability, and easy processing. Therefore, it is often used in the automotive and aerospace industries. Consequently, it is necessary to study the performance and indicators of the target materials, especially the dynamic softening mechanism and flow behavior. Furthermore, setting up a perfect constitutive prediction model of flow behavior for 5754 aluminum alloy would have far-reaching implications.

In this work, the hot compression tests were carried out on Gleeble-3500 thermo-simulation machine at different strain rates and different deformation temperatures. In order to provide accurate data and establish the model with high accuracy and stability, the temperature rise effect was considered to optimize the stress–strain data obtained. Furthermore, the experimental data and modified flow curves were applied to construct the SA model and the back propagation artificial neural network (BP–ANN) model. The use of GA led to the determination of optimal weight and threshold for ANN simultaneously. After this, the performance of the three models was compared according to the correlation coefficient (*R*) and the average absolute relative error (*AARE*). Finally, the dynamic softening mechanism of 5754 aluminum alloy was analyzed by the stress–strain curves of a single pass.

## 2. Experiment and Materials

**Figure 1:**
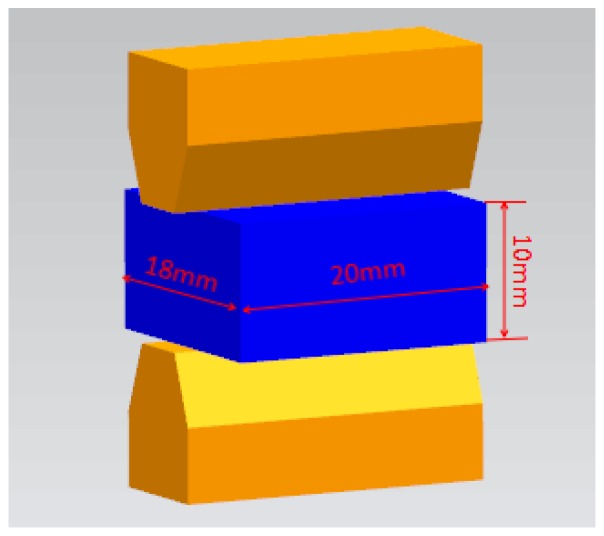
The initial shape and size of the specimens.

The hot compression tests were carried out on a Gleeble-3500 thermo-simulation machine (Dynamic System Institution, New York, NY, USA). The nominal chemical composition (wt %) of the 5754 aluminum alloy is shown in Table 1. The geometric shapes and size of the specimens for testing are shown in Figure 1.

**Figure 2:**
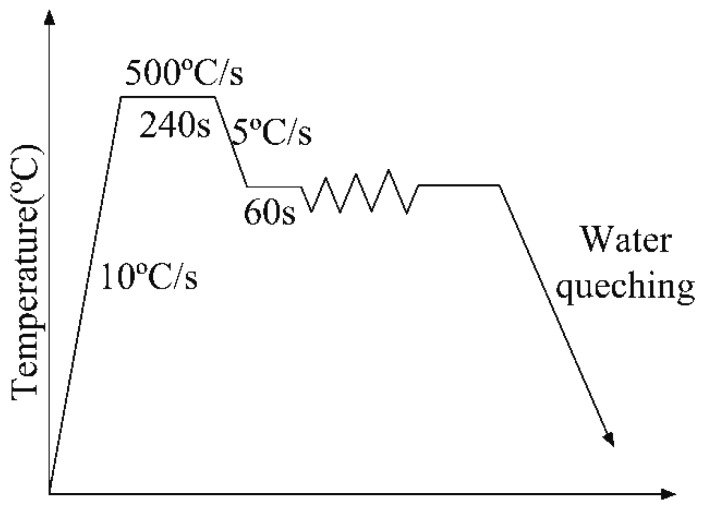
The flowsheet of hot compression tests.

First, the specimens were heated to the holding temperature (500 °C) by resistance with a heating speed of 10 °C/s and insulation for 4 min. After this, we cooled the specimens at different deformation temperatures at a rate of 5 °C/s to eliminate the thermal gradients and ensure that heating was complete before compression. The variation range of the test parameters were as follows: deformation temperatures were 300–500 °C and strain rates were 0.1–10 s^−1^. The whole process of hot compression tests ended when the compression deformation reached 70%. The specimens were quenched immediately after the experiment. The specific experimental procedure is shown in Figure 2.

## 3. Results and Discussion

### 3.1. The SA Model

According to the Arrhenius model, the constitutive relationship of the material at low stress levels can be expressed as follows:

(1) $$\dot{\varepsilon }=A_{1}\sigma^{n_{1}}\exp(-Q/RT)$$

For high stress levels, this equation becomes:

(2) $$\dot{\varepsilon }=A_{2}\exp(\beta \sigma )\exp(-Q/RT)$$

For all stress levels, the following equation can be applied:

(3) $$\dot{\varepsilon }=A_{3}{\left[\sinh(\alpha \sigma )\right]}^{n}\exp(-Q/RT)$$

where σ represents the true stress (MPa); T and $\dot{\varepsilon }$ represent the absolute temperature (K) and strain rate (s^−1^), respectively; Q and R are the activation energy (J mol^−1^) and gas constant, respectively; and α, β, $A_{1}$, $A_{2}$, $A_{3}$, $n_{1}$, and n are the material constants. α can be derived by:

(4) $$\alpha =\beta /n_{1}$$

The influence of the deformation parameter on the flow stress can be defined as *Z* (Zener–Holloman parameter):

(5) $$Z=\dot{\varepsilon }\exp(Q/RT)$$

**Figure 3:**
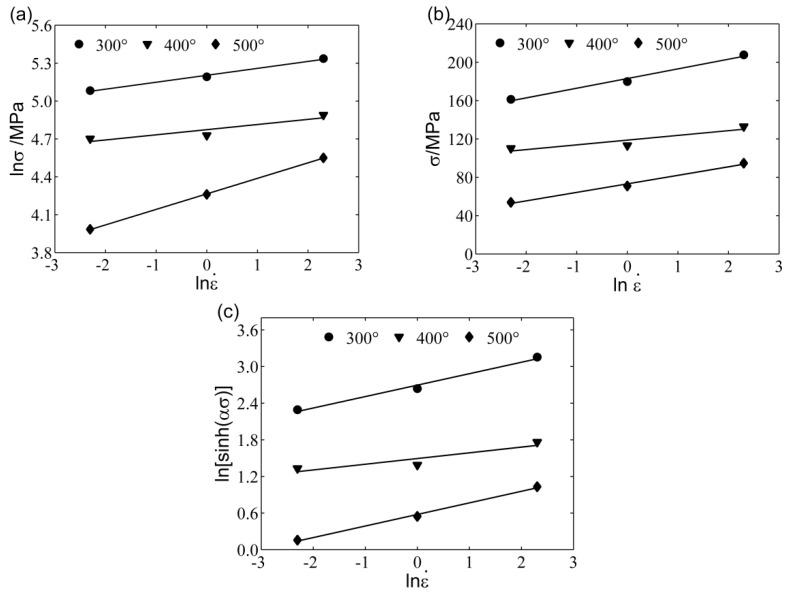
Plots between (**a**) lnσ vs. $\ln\dot{\varepsilon }$; (**b**) σ vs. $\ln\dot{\varepsilon }$; and (**c**) ln[sinh(ασ)] vs. $\ln\dot{\varepsilon }$.

The material constants were calculated at the true strain of 0.2. As shown in Figure 3a,b, there is a linear relationship between lnσ and $\ln\dot{\varepsilon }$, where σ and $\ln\dot{\varepsilon }$ are the same. Linear regression was performed using the least squares method. The reciprocals of the slopes in Figure 3a,b were used to calculate $n_{1}$ and β. After this, α can be calculated according to Equation (4). The calculated values of $n_{1}$, β, and α are 8.1376, 0.1508, and 0.0185, respectively. As shown in Figure 3c, using the same method, the value of *n* can be calculated from the mean reciprocals of the slope for the lines in the figure (*n* = 5.3029).

We assumed that Q had no correlation with temperature. Equation (3) can be converted into Equation (6):

(6) $$\ln[\sinh(\alpha \sigma )]=\frac{\ln\dot{\varepsilon }-\ln A}{n}+\frac{Q}{nR}\frac{1}{T}$$

Differentiating Equation (6) gives:

(7) $$Q=Rn{\frac{\partial \ln[\sinh(\alpha \sigma )]}{\partial (1/T)}|}_{\dot{\varepsilon }}$$

**Figure 4:**
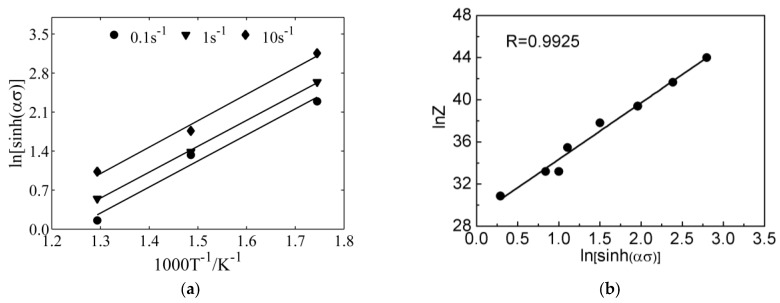
The relations between (**a**) ln[sinh(ασ)] and 1000 T^−1^; and (**b**) lnZ and ln[sinh(ασ)].

Q (206,669 J mol^−1^) can be calculated from the mean slope of the lines in Figure 4a. After this, the *Z*-parameter can be determined according to Equation (8):

(8) $$Z=\dot{\varepsilon }\exp(206669/RT)$$

Equation (9) can be derived by combining Equations (3) and (5). After this, *A* can be determined by the intercept of the line in Figure 4b (*A* = 3.93 × 1017), where:

(9) $$Z=A_{3}{\left[\sinh(\alpha \sigma )\right]}^{n}$$

The constitutive model can be obtained under various conditions by coupling the *Z*-parameter as follows:

(10) $$\sigma =\frac{1}{\alpha }\sinh^{-1}[{\left(\frac{\dot{\varepsilon }\exp(Q/RT)}{A}\right)}^{1/n}]$$

At this point, all the constitutive parameters of the material have been obtained. Further details relating to the analysis process of the material constants can be obtained from References [29,30,31]. After this, the constitutive model of the 5754 alloy at the true deformation strain of 0.2 can be determined. Using the above modeling method, the constitutive constants at other true deformation strains can be calculated. The relationship between the strain and constitutive constants can be obtained by polynomial fitting. Finally, the SA model within the test conditions can be determined. 

**Figure 5:**
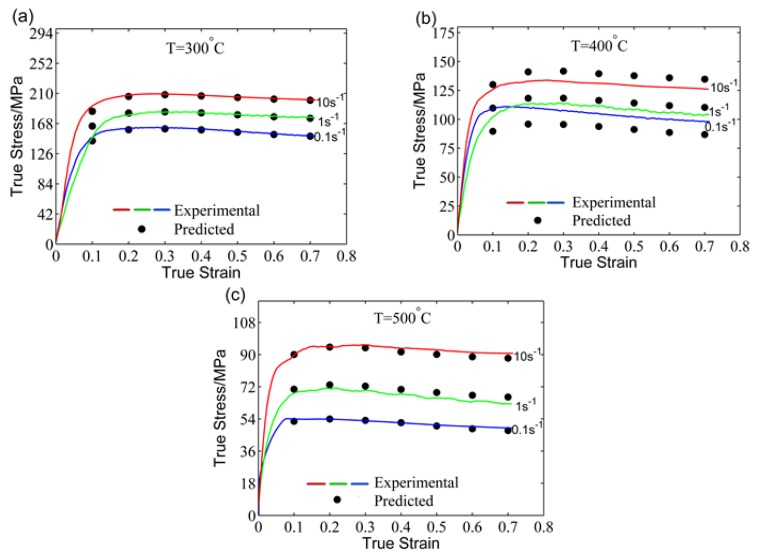
Comparisons of the predicted and experimental flow stress by the strain-compensation Arrhenius (SA) model at (**a**) 300 °C, (**b**) 400 °C, and (**c**) 500 °C.

The predicted results using the SA model under the test conditions are shown in Figure 5. Clearly, the flow behavior of the 5754 aluminum alloy can be properly predicted by this model. In addition, it can be observed that the model has a large predicted error at 400 °C (Figure 5b).

### 3.2. Correction of Temperature Rise Effect

**Figure 6:**
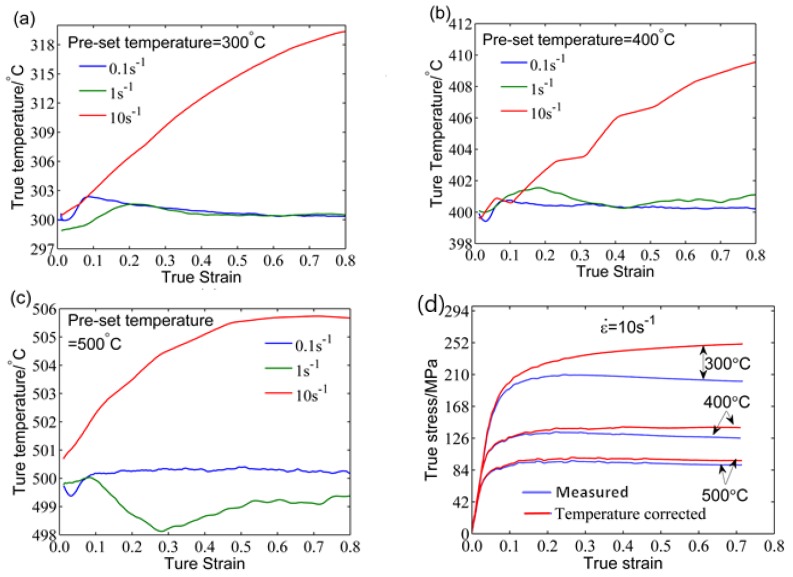
The variation in true deformation temperature according to true strain at the pre-set deformation temperatures of (**a**) 300 °C, (**b**) 400 °C, and (**c**) 500 °C; (**d**) the corrected flow curves with preset deformation temperature and a strain rate of 10 s^−1^.

During the compression deformation process, when the strain rate is less than 1 s^−1^, the change of temperature can be compensated by the heating system of the Gleeble-3500 thermo-simulation machine, with the tests being considered under isothermal compression. However, the rise in temperature of the deformation increases with increasing strain rate. When the actual deformation temperature is more than 5 °C higher than the pre-set temperature, the temperature rise effect begins to occur, which can decrease the flow stress. Correspondingly, the softening effect will increase. Figure 6a–c shows the variation in true deformation temperature compared to the true strain at the pre-set deformation temperature. It can be seen that the temperature rise effect should be corrected at a strain rate of 10 s^−1^. Similar results can be found in Reference [32]. In general, the temperature correction factor proposed by Devadas [33] can be used to correct the flow stress data, as shown by Equation (11):

(11) $$\Delta \sigma =\frac{Q_{def}}{n\alpha R}[\frac{1}{T+\Delta T}-\frac{1}{T}]$$

where $Q_{def}$, α, n, R*,* and T have the same meaning as mentioned previously, and ΔT is the absolute temperature increment (K).

As shown in Figure 6d, after correcting for the temperature rise effect, the flow stress increased significantly. The details of the temperature correction and the process of calculation can be found in References [32,34].

### 3.3. The BP–ANN Model

**Figure 7:**
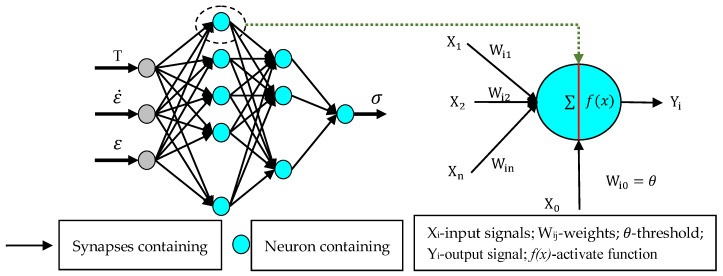
The structure of the artificial neural network (ANN) model and the neuron model.

Artificial neural network (ANN), which has several performance characteristics that are similar to neural networks in the human brain, is a large-scale parallel distributed information processing system [35]. The neurons are the basic elements of ANN, which can be used to deal with complex data information. The research shows that the performance of the ANN is roughly determined by its structure and the weights of the connections [36]. Phaniraj and Lahiri [37] and Lucon and Donovan [38] introduced the BP algorithm as a representative method to reduce the errors created by the gradient descent method. Therefore, a four-layered BP–ANN architecture model was established to determine the flow behavior of the 5754 aluminum alloy. The schematic diagram for the ANN architecture and neuron model is shown in Figure 7.

The mathematical form of the neuron model is shown in Equations (12) and (13). The deformation parameters are used as the input signals for the input layer neurons in parallel, while the flow stress is calculated as the response to the output layer of neurons. The Levenberg–Marquardt algorithm has been used to train this ANN model. The use of two hidden layers with 10 and 8 neurons, respectively, gives the best predictions. The hidden layer neurons use the hyperbolic tangent sigmoid transfer function, while the output layers use the linear function:

(12) $$net_{i}=\sum_{j=1}^{n}W_{ij}X_{j}-\theta$$

(13) $$Y_{i}=f(net_{i})$$

where $X_{j}$ represents all the input signals for the neurons; $W_{ij}$ and θ represent the weights and thresholds, respectively; $Y_{i}$ is the output signal for the neurons; and f(x) is the activate function.

The MATLAB–Neural Network toolbox was used to develop the ANN model. A total of 234 data points were obtained from the experimental data at true strains of 0–0.7 at appropriate intervals. The other data are shown in Table 2. Before establishing the ANN model, all collected data should be normalized by Equation (14) to reduce the order of magnitude difference between the various dimensions:

(14) $$X_{K}=\frac{\left(x_{k}-x_{\min}\right)}{\left(x_{\max}-x_{\min}\right)}$$

where $X_{K}$ and $x_{k}$ are the experimental data and the processed data, respectively; and $x_{\max}$ and $x_{\min}$ are the maximum and minimum values of the experimental data, respectively.

**Figure 8:**
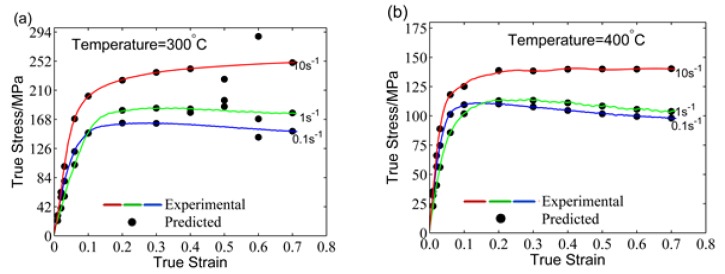

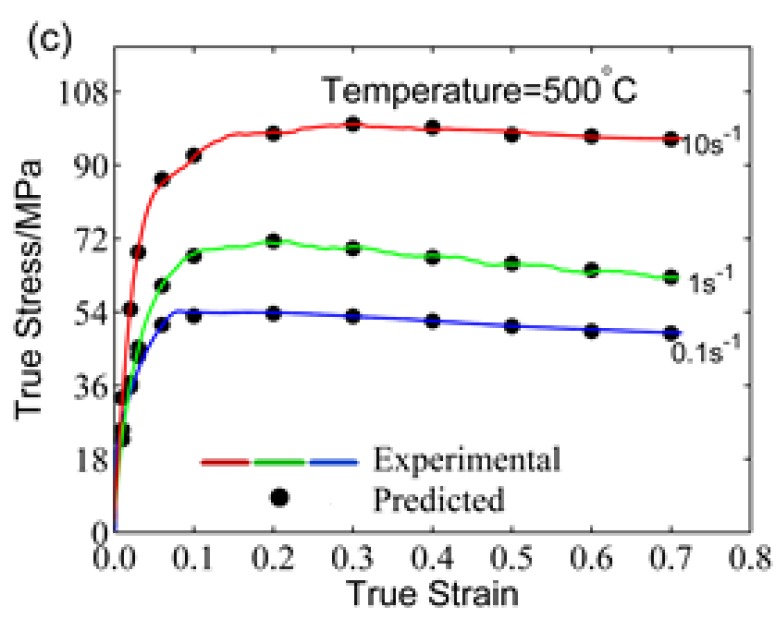
Comparisons of the predicted and experimental flow stress by the non-optimized ANN model at: (**a**) 300 °C, (**b**) 400 °C, and (**c**) 500 °C.

After the ANN training is completed, the flow stress of the 5754 aluminum alloy under test conditions can be predicted by the BP–ANN model as shown in Figure 8. The predicted values are consistent with the experimental data over the whole range except at 300 °C and true strains in the range of 0.4–0.6. As BP–ANN lacks the ability to optimize globally, this may cause the algorithm to fall into a local optimum. Through training of 100 times, we found that each training has different prediction accuracy, which means that the prediction accuracy fluctuates randomly according to the training times.

### 3.4. The ANN–GA Model

**Figure 9:**
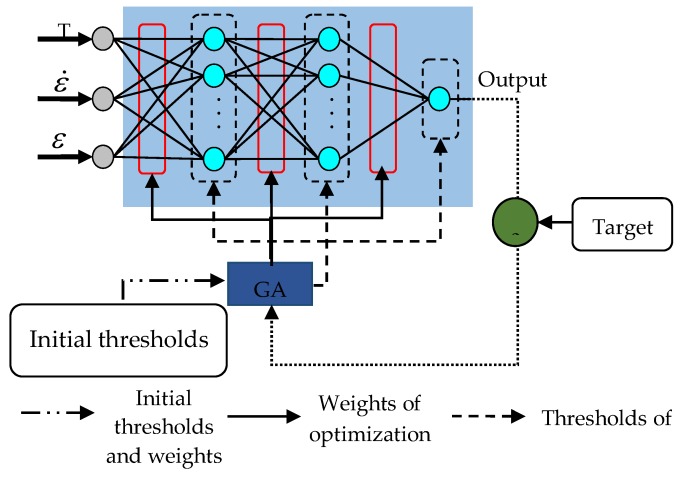
Schematic structure of the optimization using genetic algorithm (GA).

The choice of the ANN connection weights and thresholds has a great influence on the stability and training efficiency of the network. The overall distribution of the ANN weights and thresholds determines the fidelity of the model. However, the initial weights and thresholds of ANN are randomly generated by the system so that the network is not convergent, which leads to instability in ANN for the prediction of flow behavior. This paper proposes an intelligent algorithm based on the GA to optimize the BP–ANN. Figure 9 shows the optimization process of BP–ANN using GA.

Genetic algorithm (GA) is a heuristic parallel search technique based on the dynamics of natural selection and genetics, which has very good optimization ability [36]. The GA consists of a population of many individuals. Each individual has its own fitness. The GA always retains the most successful individual and eliminates the low-fitness individual to achieve the best solution. Further details about the concepts and theories of GA can be obtained from reference [39].

**Figure 10:**
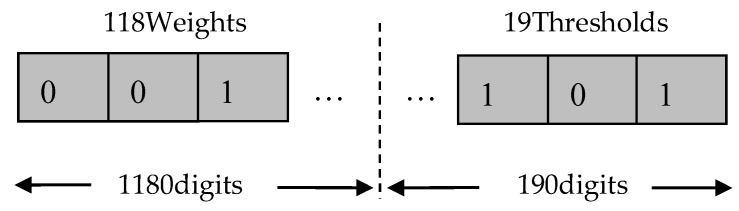
The binary coding of the neural network initial weights and thresholds.

This article used MATLAB to write all the codes for the ANN model and GA. The structure of BP–ANN has been introduced in Section 3.3. A four-layered BP–ANN architecture has been selected. The number of neurons in each layer is 3, 10, 8 and 1, respectively. There are 118 weights (3×10+10×8+8×1=118) and 19 thresholds (10+8+1=19). Therefore, GA needs to optimize 137 parameters that are connected to an individual. The individual boundary conditions are between −0.5 and 0.5. Each individual is encoded by binary methods, which is shown in Figure 10. The length of each variable in the individual is 10 digits. Therefore, each individual needs 1370 encoding lengths. Table 3 shows the parameters of GA. In order to realize the GA for optimizing the BP–ANN, the predictive error norm of the neural network is used to measure the prediction accuracy and generalization ability of the model. Therefore, the fitness values of each individual in GA are calculated by the error norm.

**Figure 11:**
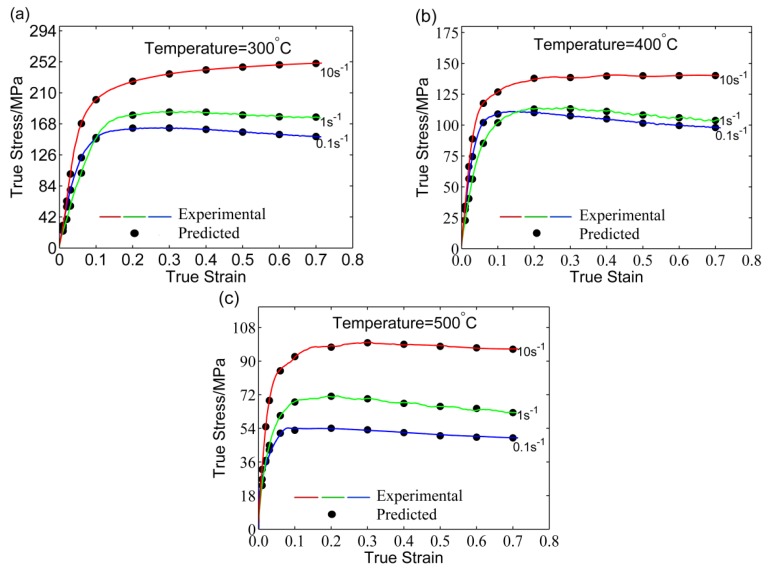
Comparison between the original and calculated stress data by ANN–GA at (**a**) 300 °C, (**b**) 400 °C and (**c**) 500 °C.

The predicted results of ANN–GA model are shown in Figure 11. The results show that the predicted values can fit the experimental value faultlessly within the whole deformation range. Finally, an ANN–GA model with high accuracy and stability has been developed.

### 3.5. Performance of the SA, BP–ANN, and ANN-GA Models

The predictability of the SA, BP–ANN, and ANN–GA models were quantified by *R* (Equation (15)) and *AARE* (Equation (16)), which are expressed as follows:

(15) $$R=\frac{\sum_{i=1}^{N}(\sigma_{O}^{i}-{\bar{\sigma }}_{O})(\sigma_{C}^{i}-{\bar{\sigma }}_{C})}{\sqrt{\sum_{i=1}^{N}{\left(\sigma_{O}^{i}-{\bar{\sigma }}_{O}\right)}^{2}\sum_{i=1}^{N}{\left(\sigma_{C}^{i}-{\bar{\sigma }}_{C}\right)}^{2}}}$$

(16) $$AARE(\%)=\frac{1}{N}\sum_{i=1}^{N}\left|\frac{\sigma_{O}^{i}-\sigma_{C}^{i}}{\sigma_{O}^{i}}\right|\times 100\%$$

where $\sigma_{O}^{i}$ is the original data; $\sigma_{C}^{i}$ is the calculated value derived from the established SA, BP–ANN, and ANN–GA models; $\sigma_{O}^{i}$ and $\sigma_{C}^{i}$ are the mean values of $\sigma_{O}^{i}$ and $\sigma_{C}^{i}$, respectively, and *N* is the amount of the experimental data.

**Figure 12:**
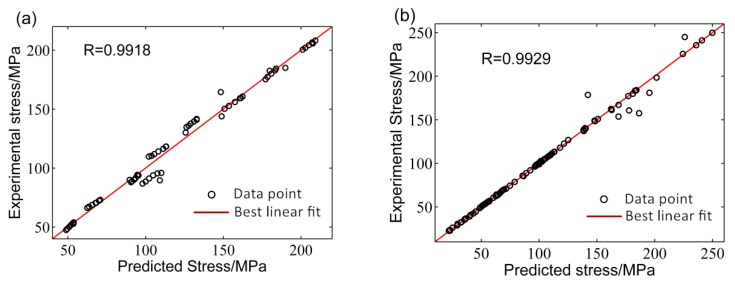

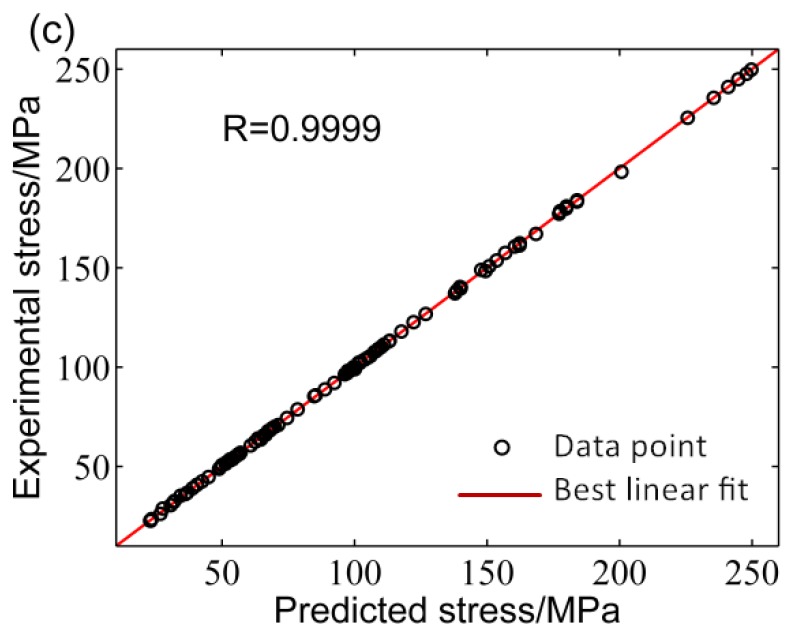
Correlation between the predicted and the experimental values of flow stress by: (**a**) SA, (**b**) BP–ANN, and (**c**) ANN–GA models.

*R* is usually applied to evaluate the linear correlation between the experimental and predicted values [14,22,26]. However, it is unable to evaluate when the prediction is biased towards a local scope. Consequently, the *AARE* was used to check the reliability of models. The *R* of the three developed models are shown in Figure 12a–c, respectively. Most data points could track the ideal regression line and achieved good correlation between the predictive and experimental values of flow stress. Moreover, the *R* for SA, BP–ANN, and ANN–GA models were 0.9918, 0.9929, and 0.9999 (Table 4). The results reflected that the developed ANN–GA had better predictive ability for the flow behavior compared to the SA and BP–ANN models. 

**Figure 13:**
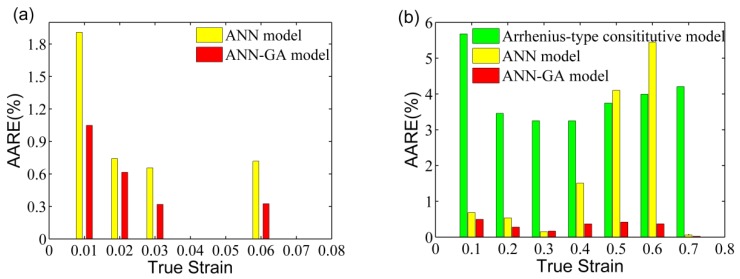
The *AARE* of the (**a**) BP–ANN and ANN–GA models at true strains of 0.01–0.06; and (**b**) Arrhenius, BP–ANN, and ANN–GA models at true strains of 0.1–0.7.

Figure 13 shows the values of *AARE* of the developed models under the test strain conditions. The *AARE*s obtained from the ANN–GA model were significantly lower than the other models in the test true strain range (Figure 13 and Table 4), which indicates that the developed ANN–GA had better stability for the prediction of flow behavior compared to other models.

After the above statistical analysis, the flow behavior of 5754 aluminum alloy could be accurately and reliably predicted by the developed ANN–GA model. In addition, the SA model contains a large number of model constants that need to be calculated repeatedly. The time it took to build the ANN model was shorter than the time required to build the phenomenological model.

### 3.6. The Dynamic Softening Mechanism of 5754 Aluminum Alloy

As shown in Figure 11, the 5754 aluminum alloy showed the typical rheological characteristics during the hot compression tests. The flow stress rose rapidly with an increase in the strain until it reached the peak. After this, the flow stress did not significantly change with increasing strain, which demonstrated a steady flow softening phenomenon. Moreover, the flow stress increased with a decrease in the deformation temperature and an increase in strain rate. It can be seen that the 5754 aluminum alloy possessed the property of strain rate dependence. In addition, the required strain for reaching the steady state deformation increased with increasing strain rate and decreasing temperature. The cross-slip of the screw dislocation and the climbing of the edge dislocation were difficult to carry out fully. Furthermore, the softening degree provided by the cross-slip and climbing was small under the conditions of a lower deformation temperature and higher strain rate, which can cause steady-state stress hysteresis.

**Figure 14:**
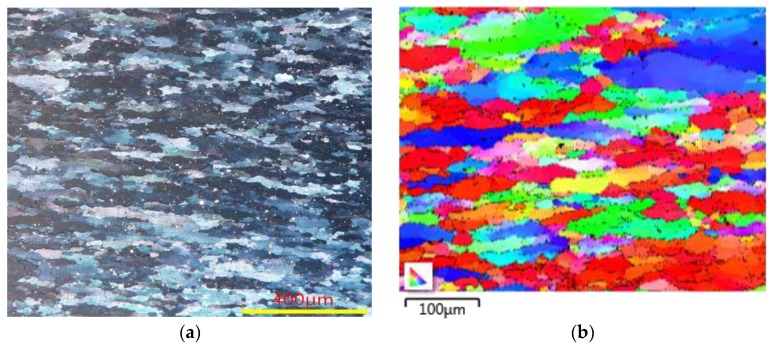
The deformed microstructures under the conditions of 500 °C and 0.1 s^−1^ using: (**a**) optical micrograph; and (**b**) electron backscattered diffraction (EBSD) mapping.

The aluminum alloy had a high stacking fault energy (SFE). Perfect dislocations were difficult to transform into partial dislocations and stacking faults, while the dynamic recovery (DRV) process required a large amount of stored energy during the process of hot deformation. Therefore, the driving force required for the dynamic recrystallization (DRX) of the alloy decreased, which increased the difficulty of DRX. The deformation microstructure of the 5754 aluminum alloy deformed at a strain rate of 0.1 s^−1^ and temperature of 500 C is shown in Figure 14. It can be seen from Figure 14a that the original cast grains were elongated after deformation. To determine the morphology of the microstructure further, a local area was selected for electron backscattered diffraction (EBSD) analysis as shown in Figure 14b. The grain shape was substantially maintained in an elongated and compressed state, and the grain boundary was relatively smooth with no remotion. Besides, no obvious equiaxed recrystallization grains were observed around the grain boundary even under the conditions of a low strain rate (0.1 s^−1^) and high temperature (500 °C). This is consistent with the dynamic softening process of the 5754 aluminum alloy as shown in Figure 11. Therefore, the dynamic softening mechanism of the 5754 aluminum alloy was mainly controlled by the DRV process during the single-pass hot compression. Further details about the dynamic softening mechanism have been reported by the authors of Reference [40].

## 4. Conclusions

An optimized constitutive model of the 5754 aluminum alloy was established to predict the flow behavior and study the dynamic softening mechanism at 300–500 °C and 0.1–10 s^−1^. The conclusions are as follows:

1. Three models were established to predict the flow behavior of 5754 aluminum alloy during the hot compression tests. The SA model had a good prediction of flow stress in the steady state for the 5754 aluminum alloy. However, it resulted in a large predicted error at 400 °C. The BP–ANN model could predict the flow curves under different conditions, but the network training results could easily find the local optimum. Therefore, the prediction results fluctuated at 300 °C and true strains in the range of 0.4–0.6. The BP–ANN model optimized by GA had the best predictive ability of the flow behavior of 5754 aluminum alloy under hot compression tests.
2. The R calculated from the SA, BP–ANN, and ANN–GA models were 0.9918, 0.9929, and 0.9999, respectively, while the *AARE* for these models were 3.2499–5.6774%, 0.0567–5.4436%, and 0.0232–1.0485%, respectively. Compared with the SA and BP–ANN models, the ANN–GA was a better predictor of the flow behavior of 5754 aluminum alloy. In addition, the ANN–GA model could find the optimal weight and threshold for ANN at the same time. Thus, the predicted values of the flow stress from the ANN–GA model were stable and the prediction accuracy was very high.
3. The 5754 aluminum alloy experienced a steady flow softening phenomenon during the hot compression tests. The flow stress rose rapidly with increasing strain until it reached a peak, before remaining constant. Besides, the flow stress and the required strain to reach the steady state deformation increased with decreasing deformation temperature and increasing strain rate. Through the observation of the microstructure, no recrystallized grains were found even at high temperature and low strain rate. Therefore, the dynamic softening mechanism of 5754 aluminum alloy was mainly controlled by the DRV process during the single-pass hot compression.

## Figures and Tables

**Table 1:** The nominal chemical composition of the 5754 aluminum alloy.

Composition	Mg	Mn	Si	Fe	Cr	Zn	Ti	Cu	Impurity	Al
Content (wt %)	2.6–3.6	0.5	0.4	0.4	0.3	0.2	0.15	0.1	0.15	Balance

**Table 2:** The parameters of the ANN model.

Total data	Training data	Verification data	Train epoch	Learning rate	Training target
234	136	98	1000	0.2	10^−7^

**Table 3:** The parameters of GA.

Encoding Length	Genetic Algebra	Population Size	Crossover Probability	Mutation Probability	Generation Gap
1370	8	5	0.7	0.01	1

**Table 4:** The obtained values of correlation coefficient (*R*) and average absolute relative error (*AARE*) for the established models.

Model	*R*	*AARE* (%)
Arrhenius	0.9918	3.2499–5.6774
ANN	0.9929	0.0567–5.4436
ANN–GA	0.9999	0.0232–1.0485
